# β-noradrenergic receptor activation specifically modulates the generation of sighs *in vivo* and *in vitro*

**DOI:** 10.3389/fncir.2013.00179

**Published:** 2013-11-12

**Authors:** Jean-Charles Viemari, Alfredo J. Garcia, Atsushi Doi, Gina Elsen, Jan-Marino Ramirez

**Affiliations:** ^1^Team P3M, Institut de Neurosciences de la Timone, UMR 7289, CNRS, Aix Marseille Univesité, Marseille, France; ^2^Center for Integrative Brain Research, Seattle Children's Research Institute, Seattle, WA, USA; ^3^Departments of Neurological Surgery and Pediatrics, University of Washington School of Medicine, Seattle, WA, USA

**Keywords:** pacemaker neurons, respiratory rhythm, norepinephrine, neuromodulation, pre-Bötzinger complex, sigh rhythmic activity, *in vivo*

## Abstract

The pre-Bötzinger complex (preBötC), an area that is critical for generating breathing (eupnea), gasps and sighs is continuously modulated by catecholamines. These amines and the generation of sighs have also been implicated in the regulation of arousal. Here we studied the catecholaminergic modulation of sighs not only in anesthetized freely breathing mice (*in vivo)*, but also in medullary slice preparations that contain the preBötC and that generate fictive eupneic and sigh rhythms *in vitro*. We demonstrate that activating β-noradrenergic receptors (β-NR) specifically increases the frequency of sighs, while eupnea remains unaffected both *in vitro* and *in vivo*. β-NR activation specifically increased the frequency of intrinsically bursting pacemaker neurons that rely on persistent sodium current (*I*_Nap_). By contrast, all parameters of bursting pacemakers that rely on the non-specific cation current (*I*_CAN_) remained unaffected. Moreover, riluzole, which blocks bursting in *I*_Nap_ pacemakers abolished sighs altogether, while flufenamic acid (FFA) which blocks the *I*_CAN_ current did not alter the sigh-increasing effect caused by β-NR. Our results suggest that the selective β-NR action of sighs may result from the modulation of *I*_Nap_ pacemaker activity and that disturbances in noradrenergic system may contribute to abnormal arousal response. The β-NR action on the preBötC may be an important mechanism in modulating behaviors that are specifically associated with sighs, such as the regulation of the early events leading to the arousal response.

## Introduction

The pre-Bötzinger Complex (preBötC) is a neural network that is critical for the generation of mammalian breathing (Ramirez et al., 1996; Tan et al., 2008; Schwarzacher et al., 2011). Isolated in transverse slices, this network continues to generate two distinct patterns of inspiratory activity that resemble those of eupnea and sigh activity (Lieske et al., 2000; Lieske and Ramirez, 2006a,b; Ruangkittisakul et al., 2008). Sighs or “augmented breaths” are large amplitude inspiratory efforts that regularly interrupt the fictive inspiratory pattern that in the intact animal represents eupnea. In the intact animal sighs maximally activate lung and chest wall mechano-receptors (Bendixen et al., 1964), and thus serve an important role in preventing atelectasis. Following vagotomy and sometimes following lesioning of the carotid sinus nerves, sighs are abolished for several hours (Bartlett, 1971; Glogowska et al., 1972; Matsumoto et al., 1997). But, sighs return after deafferentation and continue to be generated at a reduced frequency (Cherniack et al., 1981; Marshall and Metcalfe, 1988). These data suggest an important role for sensory feedback in modulating the drive to sigh but these experiments also indicate that reflexive mechanisms are not essential for their genesis. The characteristics of fictive sigh-like bursts recorded *in vitro* are consistent with the definition of sighs *in vivo* (Glogowska et al., 1972; Cherniack et al., 1981; Orem and Trotter, 1993; Takeda and Matsumoto, 1998). *In vivo* and *in vitro*, the biphasic inspiratory activity has been described as a “eupneic-triggered sigh” since a eupneic respiratory burst typically proceeds and is coupled to the sigh (Lieske et al., 2000). Under certain conditions, however, sighs can also become uncoupled from the eupneic inspiratory activity (Lieske et al., 2000). Of particular importance is the role of sighs in the events that lead to arousal (Thach, 2002). In this context, failure to sigh and arouse has been implicated in the events that ultimately lead to Sudden Infant Death Syndrome (SIDS, Kahn et al., 1988).

The action of catecholamines on respiratory activity is subtype receptors specific (Viemari, 2008; Viemari et al., 2011). Interestingly, prior reports suggest that activation of β-noradrenergic receptors (β-NR) has no significant effects on respiratory rhythmogenesis in the isolated neonatal brainstem preparation (Arata et al., 1998). Yet, the question whether different respiratory-related patterns are affected has not been addressed.

Here, we demonstrate that modulation of β-NR alters specifically sigh activity without affecting the eupneic respiratory pattern *in vivo* and in the *in vitro* medullary slice preparation. This indicates that both respiratory rhythms can be independently controlled by neuromodulators, which poses an interesting basic-scientific problem: How can these two rhythmic activities with different timing characteristics (Lieske and Ramirez, 2006a,b; Ruangkittisakul et al., 2008; Tryba et al., 2008; Koch et al., 2013) be differentially controlled by neuromodulators? This is a particularly interesting problem, since the vast majority of neurons are synaptically connected during both rhythmic activities. Specifically, all respiratory neurons recorded in the study by Lieske et al. (2000) received phasic synaptic input during both activities. Another study suggested that more than 95% of neurons were synaptically connected to both rhythms and only less than 5% of the recorded respiratory neurons received synaptic input only during the sigh (Tryba et al., 2008). Thus, these data indicate that the network(s) involved in the generation of sighing and gasping is largely overlapping.

Here, we show that β-NR activation modulates bursting pacemaker neurons that depend on *I*_Nap_, while pacemakers that depend on *I*_CAN_ remained unaffected. We also show that sighs were blocked by riluzole, an antagonist of *I*_Nap_, while they were unaffected by flufenamic acid (FFA), an antagonist of *I*_CAN_. Taken together, these data support the notion that heterogeneous cellular mechanisms differentially contribute to the generation of fictive eupneic and fictive sigh rhythms.

## Materials and methods

### The transverse slice preparation

Brainstem transverse slice preparation from CD1 mice (P6–P12) were obtained using a technique described in detail previously (Ramirez et al., 1996). The most important steps are summarized here. All surgical and experimental procedures conformed to guidelines from the French Ministry for Agriculture and Fisheries and were approved by the Institutional Animal Care and Use at the Seattle Children's Research Institute. The mice were anesthetized by hypothermia and decapitated. The isolated brainstem was then placed in ice-cold artificial cerebro-spinal fluid (a-CSF) bubbled with carbogen (95% O_2_ and 5% CO_2_). The a-CSF contained (in mM): 128 NaCl, 3 KCl, 1.5 CaCl_2_, 1 MgCl_2_, 24 NaHCO_3_, 0.5 NaH_2_PO_4_, and 30 D-glucose, pH of 7.4. The brainstem was then glued to an agar block on the mounting plate of a VT 1000 s (Leica Microsystems, Richmond Hill, ON, Canada) with the rostral end up and the ventral face toward the blade. 100 to 200 μm Hundred to two hundred micrometers serial transversal slices at a 20° angle were then made in a rostral to caudal direction until disapperance of parafacial group and appearance of the inferior olive, nucleus ambiguus, the hypoglossal nucleus, and the opening of the fourth ventricle as also described in the P0 atlas by (Ruangkittisakul et al., 2011). Then, a 550–650 μm thick a rhythmic slice containing the preBötC was made. The approach encompasses the preBötC (Figure 2). In this figure we cut the slice into three parts and stained the slices with NK1/DAPI antibodies (Figures 2C–E′). The boundaries of NK1R+ cells in the ventral respiratory column correspond to ~300 μm-thick sections in total. Note that the preBötC area shows a high concentration of NK1 staining (Figures 2C,C′) as previously described by different groups (Gray et al., 1999; Guyenet et al., 2002; Pagliardini et al., 2003). These slices also contain raphé neurons, Chx10 neurons (Figures 3, 6; Crone et al., 2012) and TH-neurons important for the stabilization of the respiratory rhythm (Viemari et al., 2005; Zanella et al., 2006).

Slices are transferred into a recording chamber, continuously superfused with oxygenated a-CSF and maintained at a temperature of 30 ± 0.5°C. The potassium concentration of the perfusate was raised from 3 to 8 mM over 30 min to ensure a long-lasting stable rhythm due to the duration of many of the protocols. It must be emphasized that a significant proportion of slices generates rhythmic activity already in 3 mM potassium (Tryba et al., 2003).

### Tissue preparation and histological analysis of preBötC

As mentioned above, slice preparations from P7 CD-1 mice (*n* = 4) were processed for tissue histology. Briefly, 550 μm transverse sections of the medulla encompassing the preBötC were fixed in cold buffered 4% paraformaldehyde (PFA) in 1× Phosphate Buffered Saline (PBS) overnight at 4°C, frozen in optimum cutting temperature compound (OCT, VWR International, Radnor, PA, USA), cryostat sectioned at 14 μm (for Nissl stain and immunofluorescence), and mounted on Superfrost Plus slides (Thermo Fisher Scientific, Waltham, MA, USA). Slide-mounted sections were stored at −80°C until needed. For Nissl staining, 14 μm sections were stained with 0.5% cresyl violet, as previously described (Hevner et al., 2001). Immunofluorecence staining was done as previously described (Bedogni et al., 2010). Briefly, cryosections were air dried, washed three times in 1× PBS, blocked for 1 h at room temperature (RT) with 5% goat serum in PBS containing 0.3% Triton-X 100 and 0.2% bovine serum albumin (blocking solution) and then incubated overnight at 4°C with rabbit polyclonal anti-NK1R antibody (Advanced Targeting Systems, San Diego, CA, USA; 1:500). Species-specific fluorescent-tagged secondary antibody (Molecular Probes/Life Technologies, Grand Island, NY, USA; Alexa-Fluor-568 at 1:400 dilution) was applied for 2 h at RT, sections were counterstained with the nuclear label DAPI (0.01%, Molecular Probes/Life Technologies, Grand Island, NY, USA) and coverslipped with microscope cover glass (Thermo Fisher Scientific, Waltham, MA, USA) using Fluormount-G (Southern Biotech, Birmingham, AL, USA). Mosaic images of Nissl stain and bright field live images at low magnification were obtained using a Zeiss Axioimager Z1 microscope with Axiovision v4.7 software (10× objective). Fluorescent images detecting NK1R antibody in the preBötC at high magnification were obtained using a Zeiss LSM 710 confocal microscope (40× objective, 543 nm laser line).

### Drugs and solutions

A cocktail of antagonists for NMDA-receptors [CPP-(RS) 10 μM, Tocris Cookson, Ellisville, MO], non-NMDA receptors (CNQX 20 μM, Tocris Cookson), glycine-receptors (strychnine 1 μM, SIGMA-RBI, St. Louis, MO) and GABA_A_-receptors (bicuculline-free base 20 μM, SIGMA-RBI) was used to block fast synaptic transmission (Peña et al., 2004). Bicuculline free base used in the present study has a very different pharmacology than the commonly used bicuculline salts (e.g., bicuculline methiodide), and the free base does not block apamin-sensitive calcium-activated potassium currents (Seutin and Johnson, 1999). To block either *I*_*Nap*_ or *I*_CAN_, we bath-applied riluzole hydrochloride (riluzole 20 μM, Tocris Cookson, and SIGMA-RBI) or FFA (50–500 μM, SIGMA-RBI), respectively. We used a wide range of FFA concentration since FFA is known for its non-specific actions (Guinamard et al., 2013). All drugs were initially solubilized in dimethylsulfoxide (DMSO, SIGMA-RBI). Norepinephrine (NE; Arterenol hydrochloride), prazosin hydrochloride (antagonist of α1-noradrenergic receptors; α1-NR), yohimbine hydrochloride (antagonist of α2-noradrenergic receptors; α2-NR), isoproterenol hydrochloride (agonist of β-NR) or propanolol (antagonist of β-NR) (SIGMA-RBI) was added to ACSF. Each drug was applied only once in a given slice, and only one slice was obtained per animal.

### *In vivo* anesthetized mouse preparation

CD1 mice (P9–P12) were anesthetized with urethane (1.5 g/kg). To characterize the respiratory activity of these freely breathing mice, we obtained electromyography (EMG) recordings from intercostal muscles. The mice were placed in a supine position and the head was fixed within a stereotaxic apparatus. The neck of the mice was opened from the ventral side, the trachea was cut and a plastic Y-shaped tubing for supplying oxygen was inserted into the proximal end of the trachea (“cannulation”). The bone of the skull covering the ventral brainstem was partially removed. The dura and arachnoid membrane were removed to expose the ventral medulla. The surface of the ventral medulla was continuously superfused with 95% O2–5% CO2 equilibrated aCSF solution at 30 ± 0.5°C. In all cases, 100% oxygen was supplied through the cannulation to avoid the need for artificial ventilation (Doi and Ramirez, 2010).

### Microinjection studies in *in vivo* anesthetized mice.

Microsyringes (Hamilton microsyringe no. 80330) with 33 gauge needles containing NE agonist 30 μM isoproterenol, total 0.6 μl) were positioned with micromanipulators (KITE, World Precision Instruments). The needles of the microsyringes were inserted into the right preBötC from the ventral side. During hypoglossal nerve recording or intercostal EMG recordings, these antagonists were microinjected into the right preBötC area at a rate of 0.3 μl/min. We did not attempt to perform bilateral needle injections to limit the damage to the preBötC, which would have compromised respiratory rhythm-generating mechanisms. Identification of drug injection site has been performed as previously described (Doi and Ramirez, 2010). Control experiments were done and injection of aCSF (~300–400 nL/min) had no effect on respiratory stability or patterns (*n* = 4, not shown).

### Extracellular recordings

**Figure 1:**
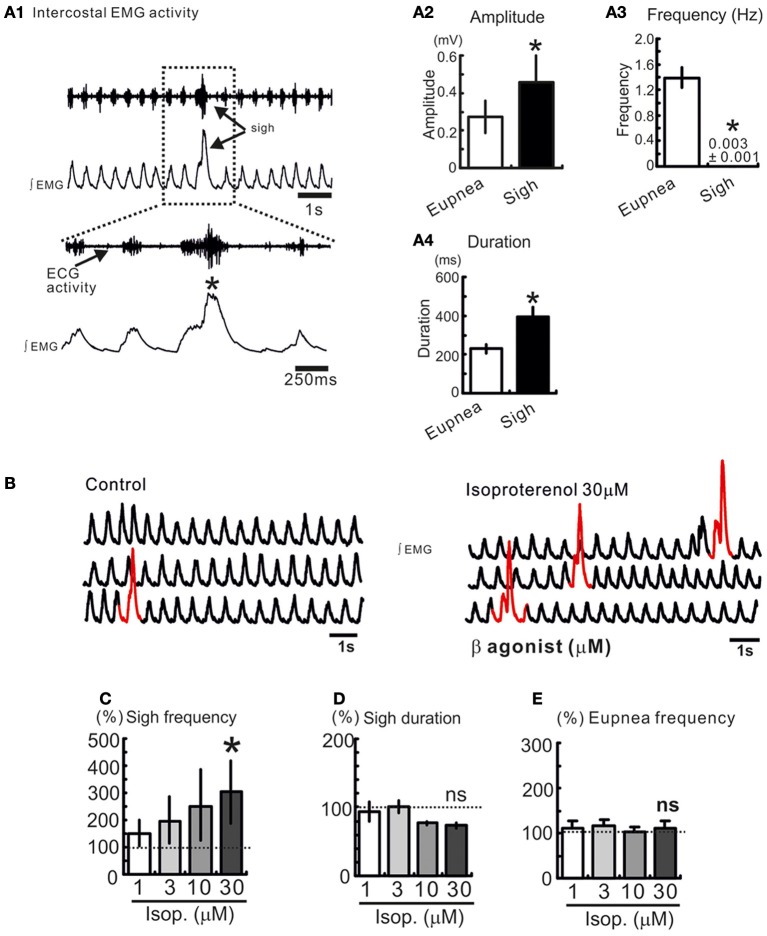
**Sighs are spontaneously generated in freely breathing mouse preparation. (A1)** Intercostal EMG activity recording in an anesthetized freely breathing mouse. Sigh is generally triggered and preceded by an inspiratory burst of fictive eupneic activity. Histograms summarize the differences between spontaneous sighs and fictive respiratory activity, in amplitude **(A2)**, frequency **(A3)**, and duration **(A4)**. (^*^p < 0.05, *n* = 8). **(B)** Trace of integrated intercostal EMG activity (∫EMG) during control and after injection of isoproterenol into the preBötC (30 μM). **(C–E)** Histograms show the effects of isoproterenol on sigh frequency **(C)** duration **(D)** and on eupnea frequency **(E)** (ns: not significant, ^*^p < 0.05, *n* = 4–8).

In the transverse slice preparation population activity recordings were obtained with suction electrodes positioned on the surface of the slice in the area including the preBötC. The slice preparation is placed rostral side up and encompasses network components rostral to the preBötC that appear to be critical for generating the sigh rhythm in very thin slices (Ruangkittisakul et al., 2008). The signals were amplified 2000 times, filtered (low pass 1.5 KHz, high pass 250 Hz), rectified and integrated using an electronic filter (time constant of 30–50 ms). Integrated population activity from the ventral respiratory group (VRG) was always in phase with integrated inspiratory activity of the hypoglossal motor nucleus (Telgkamp and Ramirez, 1999). Therefore, it was used as a marker for inspiratory population activity (Figure 1A). All recordings were stored on a personal computer using AxoTape (Version 2.0, Axon Instruments, Union City, CA) and analyzed offline using customized analysis software written with IGOR Pro (Wavemetrics, Lake Oswego, OR). Bursts were automatically detected by the IGOR program as described extensively in our previous study (Tryba et al., 2003; Viemari and Ramirez, 2006).

### EMG recordings

Eupneic activity was characterized with rostral intercostal muscles EMG recordings. For this purpose we used teflon-coated Ag bipolar electrode. The teflone coating was removed at the recording surface of this electrode. The skin covering the abdominal and intercostal muscles on the right side was partially removed, and the bipolar electrode was placed on the surface of the intercostal muscles. Signals were AC amplified and band pass filtered (8–3 kHz) (Doi and Ramirez, 2010).

### Intracellular recordings

A single intracellular recording was made from an inspiratory preBötC neuron (one neuron per slice) using the blind patch-clamp recording method. Inspiratory neurons are first identified in the cell-attached mode, which reveals their discharge pattern in phase with population activity. The slice preparation is placed rostral side up and we recorded neurons that are located between 100 and 300 μm from the surface, nor more superficial than 100 μm to obtain neurons that are maximally connected within the respiratory network. Experiments were then performed in whole-cell configuration with the neuron recorded in current-clamp where holding current was 0 pA. It must be emphasized that we have previously demonstrated that the whole-cell configuration does not alter the firing pattern of the recorded neuron (Peña et al., 2004). The patch electrodes are pulled from filamented borosilicate glass tubes (G150F-4; Warner Instruments, Hamden, CT) and filled with a solution containing 140 mM K-gluconic acid, 1 mM CaCl^*^_2_ 6H_2_O, 10 mM EGTA, 2 mM MgCl^*^_2_ 6H2O, 4 mM Na_2_ATP, and 10 mM HEPES. The composition of this intracellular solution and the lack of adverse effects on pacemaker activity was first demonstrated by our laboratory (Peña et al., 2004) and later reproduced by others (Del Negro et al., 2005). The K-gluconic acid-containing electrode solution resulted in a significant liquid junction potential (12 mV), which affected measured membrane potentials. All membrane potential values were corrected for this liquid junction potential as described by (Neher, 1992).

### Evoked synaptic transmission

Patch clamp recordings were made from rhythmically active preBötC neurons identified in a-CSF with 8 mM KCl. Once a stable recording was established, the circulating media was switched from 8 to 3 mM KC1 a-CSF where rhythmically bursting neurons became quiescent. To assess changes in synaptic transmission the contralateral VRG was stimulated using bipolar stimulation (100–300 μA; 150–450 μs; isolation unit, WPI, Sarasota, FL) to evoke synaptic transmissio prior and during drug application. Only evoked excitatory postsynaptic potentials (EPSPs) with a latency <10 ms were accepted for analysis.

### Statistical analysis

The data were analyzed using the so called SPSS software (SPSS Inc. Science Software, Chicago, IL). The comparison between the fictive eupneic activity and the fictive sigh activity was assessed by Mann and Whitney tests. The burst duration, amplitude, area, and frequency changes that were induced by pharmacological manipulation were assessed by a Wilcoxon ranked test. In other cases such as the FFA + NE experiments, a Friedman test was used for repeated measures in the same subjects, followed by a Dunn's tests as multiple-comparisons procedure. Statistical significance was assumed if *p* < 0.05. Deviations from the mean are given in SE.

## Results

### Activation of β-NR increases the number of sigh *in vivo*

We recorded respiratory activity in freely breathing mice under *in vivo* conditions and investigated whether β-NR activation had an effect on the different breathing patterns. Sighs are characterized by their large amplitude event that are triggered by a smaller eupneic event and are followed by a respiratory pause (“post-sigh apnea,” Figures 1A1,A2). Sighs occur at a low frequency and have a longer duration compared with eupneic events (^*^p < 0.05, Mann and Whitney test, Figures 1A3, A4). We injected isoproterenol (1, 3, 10, and 30 μM) unilaterally into the pre-BötC (Figure 1B). Isoproterenol at 30 μM significantly enhanced the sigh frequency (^*^p < 0.05, Friedman test, Figures 1B–D) compared to control, but had no effect on the eupnea frequency (Figure 1E). Isoproterenol had no effect on the different patterns while used at a concentration lower than 30 μM suggesting that no effect was due to pressure injection.

### Activation of β-NR enhances fictive sigh rhythmic activity in the transverse slice preparation

**Figure 2:**
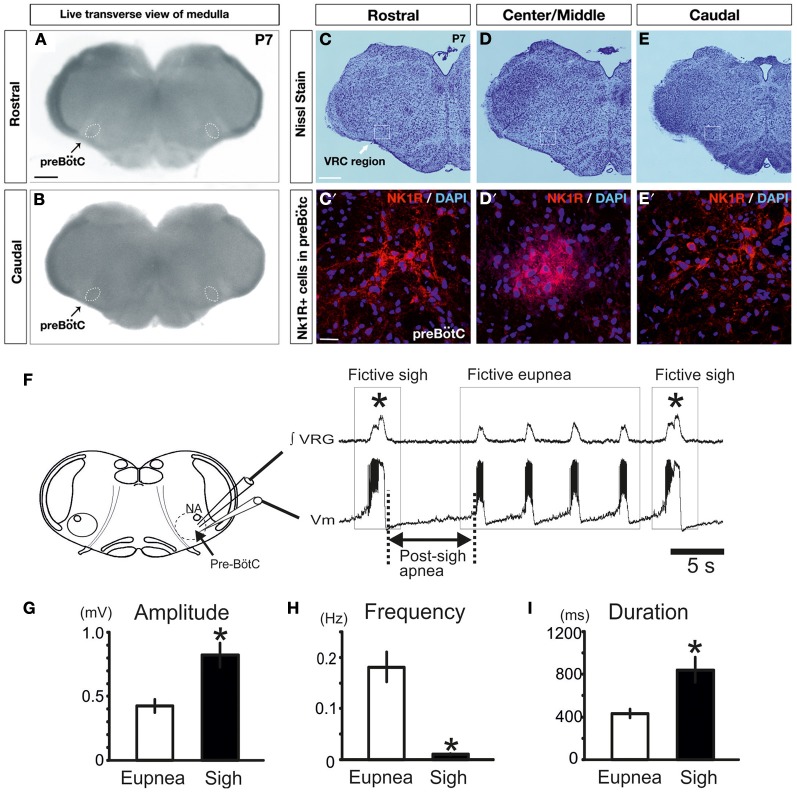
**Fictive sigh activity is observed in the *in vitro* transverse slice preparation.** Anatomical characterization of the transverse slice preparation (P7 mouse): **(A)** rostral and **(B)** caudal surface of the same live transverse slice preparation. This representative slice preparation was cut into three sections and Nissl staining was used to characterize the rostral **(C,C′)** and caudal surface **(E,E′)**, as well as the Center/Middle portion of the slice **(D,D′)**. NK1+ and DAPI+ immunoreactive neurons are depicted in **(C′–E′)**. Note that NK1R staining, which is indicative of the preBötC, is most abundant in the center, but NK1R staining extends also into the rostral and caudal portions of the slice. **(F)** Schematic of the brainstem slice preparation including the anatomical landmarks of the preBötC and recording sites of integrated VRG activity (∫VRG upper trace) and whole-cell patch clamp recordings (membrane potential, Vm, lower trace). Both traces depict fictive eupneic activity and fictive sigh activity recorded from a slice. Sighs are typically followed by a post-sigh apnea. Note that fictive sigh bursts occurred spontaneously at a slower frequency than fictive respiratory activity. Histograms summarize the significant differences between spontaneous sighs and fictive respiratory activity, in burst amplitude **(G)**, in burst frequency **(H)** and burst duration **(I)**. Results are expressed as mean ± SE. Asterisk (^*^) shows significant differences. (^*^p < 0.05, *n* = 18).

As previously shown, the medullary respiratory network isolated within the medullary transverse slice preparation *in vitro* in mice (see Figures 2A–E, and methods for details) generates under control conditions two distinct types of fictive respiratory activities: eupneic inspiratory and sigh activity as defined by (Lieske et al., 2000; Ruangkittisakul et al., 2008; Tryba et al., 2008). Fictive sigh bursts occurred spontaneously (Figure 2F) at a frequency of 0.01 ± 0.001 Hz, which is slower than the frequency of fictive eupneic inspiratory activity 0.18 ± 0.03 Hz (*n* = 18, *P* < 0.0001, Mann and Whitney test, Figure 2H). Fictive sighs have a bi-phasic shape (Figure 1A1). They are larger in amplitude (205 ± 22% of control; *p* < 0.001, Mann and Whitney test, Figure 2G), and longer in duration (198 ± 20% of control, *p* = 0.0011, Mann and Whitney test, Figure 2I) than the bursts associated with fictive eupneic inspiratory activity.

**Figure 3:**
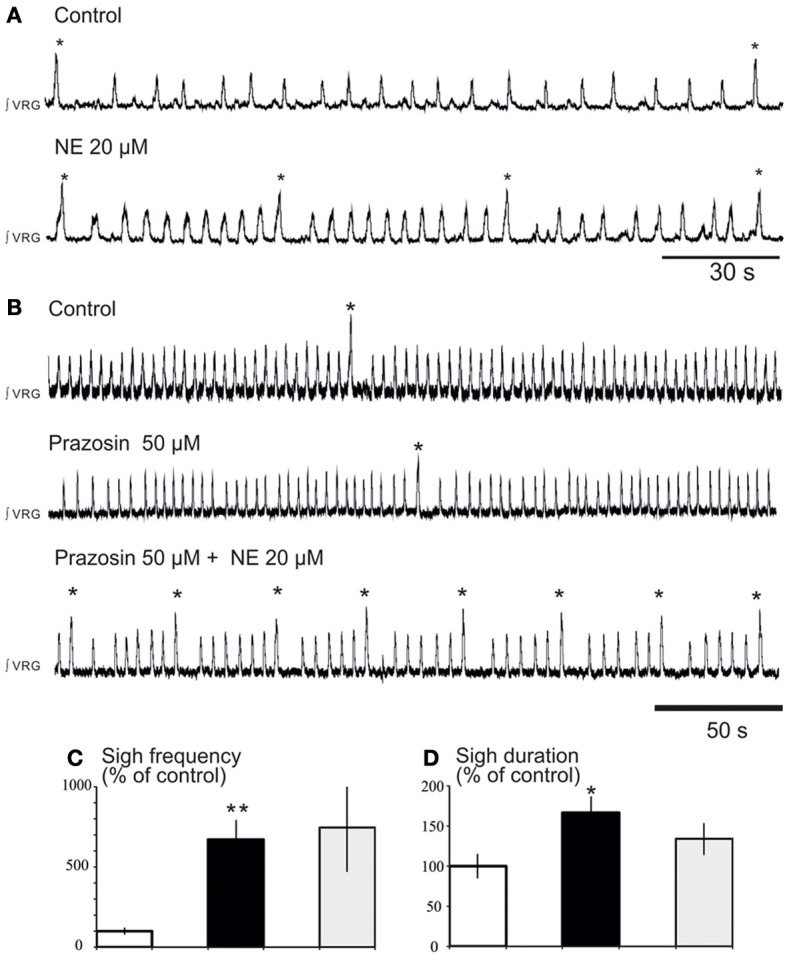
**Blockade of α1-NR does not abolish the noradrenergic modulation of fictive sigh activity. (A)** Application of NE 20 μM increases the frequency of “fictive eupneic” respiratory activity as well as the sigh activity compared to control. **(B)** Blockade of α1-NR(prazosin 50 μM) abolishes the NE-induced increase in frequency of the fictive eupneic activity but not the increased in frequency of the sigh activity. **(C,D)** Histograms show the effects of NE + prazosin on sigh burst frequency **(C)** and the sigh burst duration **(D)** (^*^p < 0.05, *n* = 4; ^**^p < 0.01).

**Figure 4:**
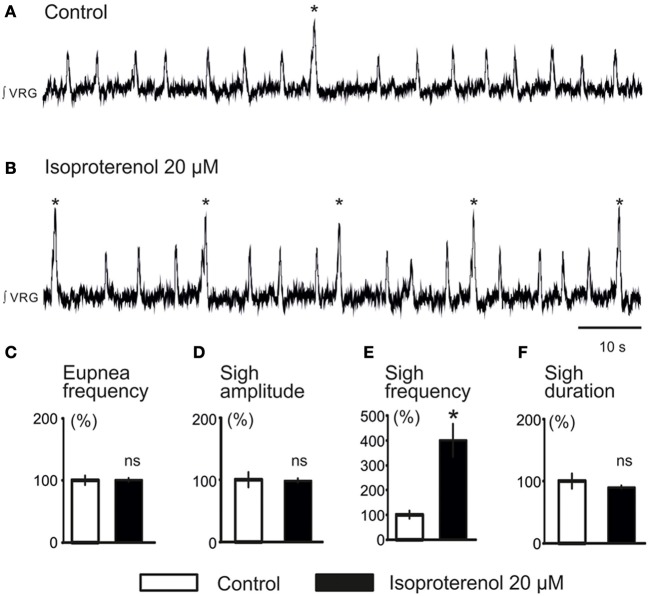
**Activation of β-NR modulates fictive sigh. (A)** ∫VRG activity recorded from a slice under control conditions. **(B)** Isoproterenol 20 μM (β-NR agonist) activates β-NR and increases specifically the frequency of sigh activity. **(C–F)** Histograms show the effects of isoproterenol on sigh burst frequency **(E)** without affecting respiratory activity **(C)**, sigh burst amplitude **(D)**, or the sigh burst area **(F)** (^*^p < 0.05, *n* = 10).

We previously reported that NE plays a major role in modulating respiratory rhythmogenesis (Viemari et al., 2004; Viemari, 2008). As illustrated in Figure 3A, NE enhanced the frequency of fictive inspiratory activity, but also sigh like activity (670 ± 120%, *n* = 7, *P* < 0.001, Friedman test). Application of the α1-NR antagonist prazosin (50 μM) prior to the NE application had no effect on baseline eupnea as previously reported (Viemari and Ramirez, 2006) and on baseline sigh like frequency. Moreover, prazosin did not affect the NE-mediated increase in sigh frequency (745 ± 270%, *n* = 4, Friedman test, Figures 3B,C) and duration (Figure 3D) suggesting that the NE-induced increase in sigh activity frequency was not mediated by α1-NRs. Similarly, application of the α2-NR antagonist yohimbine did not block the sigh-increasing effect of NE as sigh frequency was still increased (*n* = 3, data not shown). Application of the β-NR agonist isoproterenol (20 μM) significantly increased the fictive sigh frequency by 400 ± 65% (*n* = 10, *P* = 0.0022, Wilcoxon rank test, Figures 4A,B,E) without affecting the fictive eupnea frequency (*P* = 0.94, Figure 4C), the sigh amplitude (*P* = 0.28, Wilcoxon rank test, Figure 4D) or the sigh duration (*P* = 0.62, Wilcoxon rank test, Figure 4F). Similarly, the duration of the fictive post-sigh apnea was not different in the presence of isoproterenol (5.9 ± 0.07 s vs. 6.1 ± 0.1 s, *n* = 12, *P* = 0.63, Wilcoxon rank test). Application of the β-NR antagonist propranolol (50 μM, *n* = 4.4) prior to applying isoproterenol blocked the effect on fictive sighs, confirming that the modulation of sigh frequency involved specifically the activation of the β-NR (data not shown). From these results we conclude that β-NR preferentially modulate the fictive sigh rhythm.

### Isoproterenol did not significantly affect excitatory synaptic transmission between inspiratory neurons

**Figure 5:**
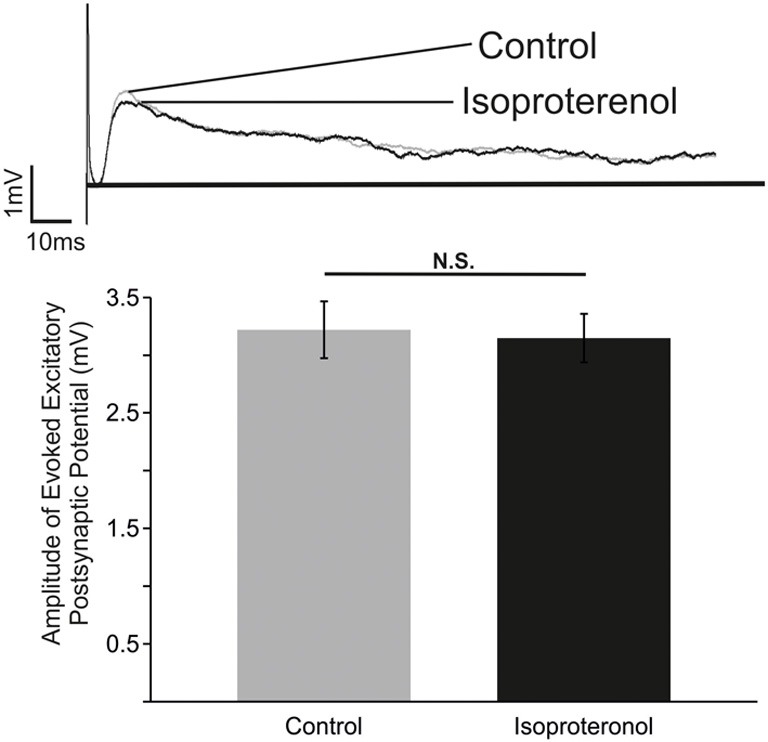
**β-NR modulation does not alter the strength of evoked EPSP in preBötC neurons. Top:** Representative traces of an electrically evoked EPSP from an preBötC neuron prior to (Control) and during application of isoproterenol (Isoproterenol). **Bottom**: Summary of the effect of isoproterenol (20–25 mM) on the amplitude of the evoked EPSP (N.S. *P* > 0.05; *n* = 5).

In an attempt to unravel the cellular mechanisms that underlie the action of β-NR, we investigated the effects of isoproterenol on evoked excitatory synaptic transmission between inspiratory neurons using methodology as previously used by Lieske and Ramirez (2006a). Neurons activated by the contralateral stimulation correspond to monosynaptically connected, glutamatergic preBötC neurons (Bouvier et al., 2010). Isoproterenol had no significant effects on the evoked EPSPs within the preBötC (Figure 5; *n* = 5). Although, these experiments cannot exclude the possibility that other, unexplored, connections were affected by isoproterenol, our data suggest that the principal effect of β-NR is not mediated by a general change in excitatory synaptic transmission.

### Isoproterenol preferentially modulates cadmium-insensitive pacemaker neurons

**Figure 6:**
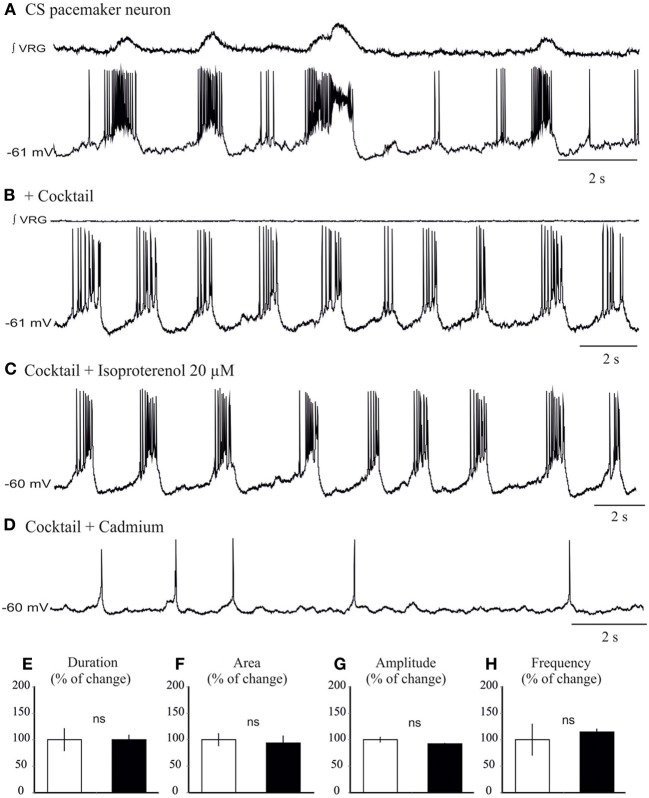
**Isoproterenol does not affect the bursting properties of *I*_CAN_ inspiratory pacemaker neurons. (A)** Recording of an inspiratory pacemaker neuron that bursts during fictive sigh activity when embedded in the network. **(B)** This neuron continues to burst intrinsically in presence of a cocktail that blocks excitatory and inhibitory synaptic transmission (See Methods). **(C)** Application of isoproterenol 20 μM does not significantly change the bursting properties of the *I*_CAN_ pacemaker neurons. **(D)** Application of cadmium 200 μM abolishes specifically bursting properties of this pacemaker neuron. Note the neuron continues to spontaneously generate action potentials **(E–H)** Histograms show no effects of isoproterenol on burst duration **(E)**, area **(F)**, burst amplitude **(G)**, and burst frequency **(H)**. These effects were quantified by obtaining for each pacemaker neuron the average burst duration, area, and frequency from 15 successive cycles (ns: not significant, *n* = 4).

We next explored the modulatory effect on neurons that possess pacemaker bursting properties (Ramirez et al., 2011; Carroll and Ramirez, 2013). In the preBötC region, two types of inspiratory bursting pacemaker mechanisms can be discriminated based on their responses to the general calcium channel blocker cadmium, (*I*_CAN_ or Cadmium-sensitive, and *I*_Nap_ or Cadmium-insensitive pacemakers, Thoby-Brisson and Ramirez, 2001; Peña et al., 2004; Viemari et al., 2011). All *I*_CAN_ and *I*_Nap_ pacemaker neurons (*n* = 10) burst during both fictive eupneic and fictive sigh activity *in vitro* when embedded in the inspiratory network (Lieske et al., 2000, Figures 6A, 7A). Isoproterenol had no significant effects on the membrane potential of *I*_CAN_ pacemaker neurons (*P* = 0.25). Moreover, in all examined, synaptically isolated, *I*_CAN_ pacemaker neurons bursting was unaffected by isoproterenol (20 μM, *n* = 4, Figure 6). No effect was observed on burst amplitude (34.6 ± 2.7 vs. 33.2 ± 3.2 mV, Figure 6E), burst frequency (0.33 ± 0.1 vs. 0.36 ± 0.1 Hz, Figure 6F), burst duration (0.98 ± 0.2 vs. 1.02 ± 0.3 s, Figure 6G) and burst area (29.35 ± 1.56 vs. 27.15 ± 1.5, Figure 6H), suggesting that *I*_CAN_ pacemaker neurons are unlikely involved in the modulation or the generation of the fictive sigh activity *in vitro*.

**Figure 7:**
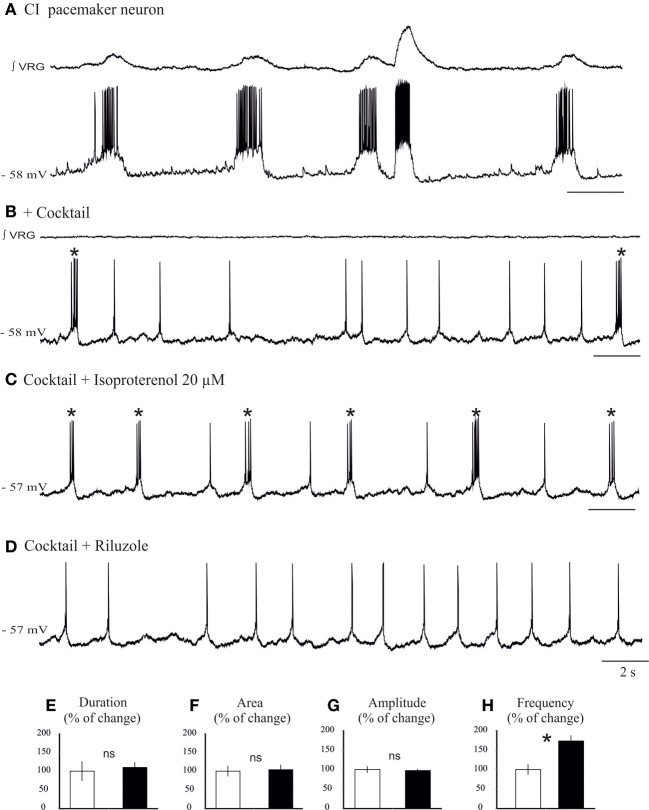
**Isoproterenol increases the burst frequency of *I*_Nap_ pacemaker neurons. (A)** Recording of an inspiratory pacemaker neuron that bursts during fictive sigh activity. **(B)** This neuron continues to burst in synaptic blockade (cocktail). **(C)** Application of isoproterenol 20 μM increases the burst frequency of *I*_Nap_ pacemaker neuron. **(D)** Application of riluzole 20 μM abolishes the bursting properties of this neuron, but the neuron continues to generate action potentials. Histograms summarize the effects of isoproterenol on burst duration **(E)**, burst area **(F)**, burst amplitude **(G)**, and burst frequency **(H)**. Note only burst frequency was significantly affected (ns: not significant, ^*^p < 0.05, *n* = 6).

We next investigated the effect of application of isoproterenol on synaptically isolated *I*_Nap_ pacemaker neurons (Figures 7B,C,D). In all *I*_Nap_ pacemaker neurons tested (*n* = 6) isoproterenol significantly increased the burst frequency (0.26 ± 0.1 vs. 0.44 ± 0.1 Hz, 173 ± 13% of control; *P* = 0.015, Wilcoxon rank test; Figure 7F), but not burst amplitude (96 ± 4% of control; Figure 7E), burst duration (106 ± 12% of control; Figure 7G) and burst area (104 ± 12% of control; Figure 7H). Further, isoproterenol induced a 1.33 ± 0.13 mV depolarization in *I*_Nap_ pacemaker neurons (data not shown). This specific modulatory action suggests that *I*_Nap_ pacemaker neurons may contribute to the modulation of the fictive sigh activity.

### Riluzole application in the network abolishes the fictive sigh rhythm

**Figure 8:**
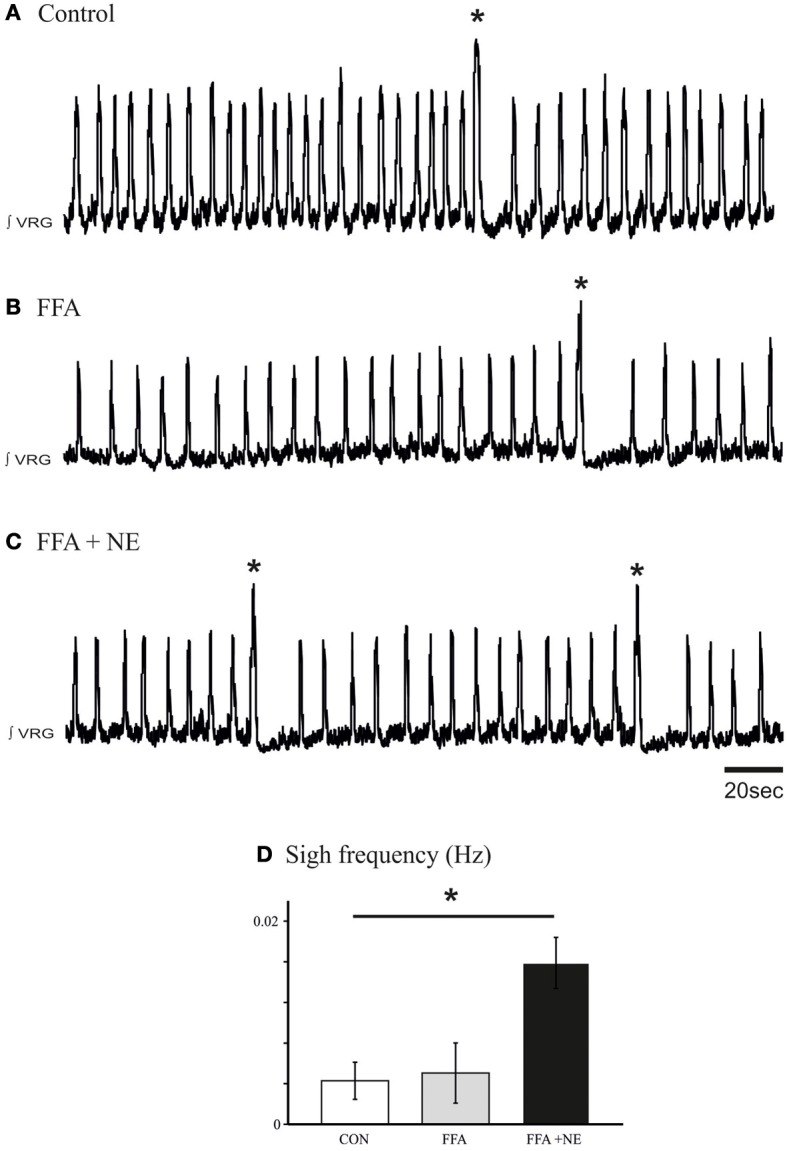
**Blockade of *I*_CAN_ with FFA does not block the β-noradrenergic modulation of sigh activity. (A)** Fictive sigh activity recorded in a control medullary slice preparation. **(B)** Application of FFA (50–500 μM) does not affect the frequency of sigh activity compared to control. **(C)** FFA does not prevent the effect of NE on the frequency of sigh activity. **(D)** Histograms summarize the effects of NE after application of FFA on sigh activity (ns: not significant, ^*^p < 0.05, *n* = 5).

Prior studies demonstrated that the *I*_CAN_ bursting mechanism is sensitive to FFA (50 – 500 μM, Peña et al., 2004; Viemari and Ramirez, 2006; Hill et al., 2011), while the *I*_Nap_ bursting mechanism is sensitive to riluzole (20 μM; Peña et al., 2004). To test the potential involvement of these two types of bursting mechanisms in mediating the noradrenergic modulation at the network level, we blocked *I*_CAN_ with FFA (50 μM; Peña et al., 2004; Hill et al., 2011). Application of FFA (30 min, 50 μM) alone did not block the generation of spontaneous sigh like activity compared to control (Figures 8A,B), and NE still increased the sigh frequency (*n* = 9, *P* < 0.01, Friedman test, Figures 8C,D). This effect was mediated by the activation of the β-NRsince application of propanolol (25 μM, *n* = 5, data not shown) abolished the increase in sigh frequency.

**Figure 9:**
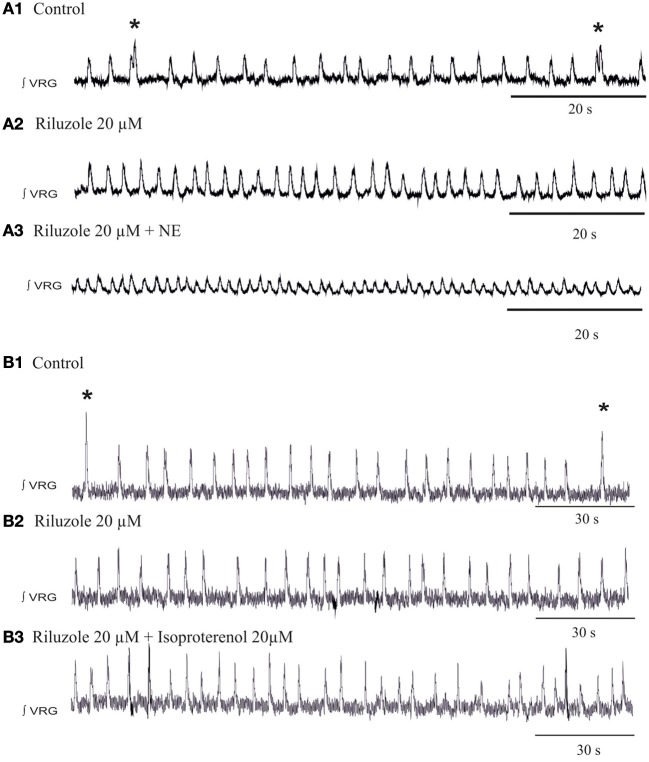
**Blockade of the *I*_Nap_ blocks the β-noradrenergic modulation of sigh activity. (A1)** ∫VRG activity recorded from a slice under control conditions. **(A2)** Application of riluzole (20 μM) abolished the generation of sigh activity. **(A3)** Riluzole did prevent the effects of NE on the frequency of sigh activity. **(B1)** ∫VRG activity recorded from a slice under control conditions. **(B2)** Application of riluzole (20 μM) abolished the generation of sigh activity. **(B3)** Riluzole did block the effect of isoproterenol (20 μM) on fictive sigh activity.

To explore the potential involvement of *I*_Nap_ in the generation of spontaneous sigh activity, we applied riluzole at concentrations that also block bursting in *I*_Nap_-dependent pacemaker neurons as previously described (Del Negro et al., 2002, 2005; Peña et al., 2004; Viemari and Ramirez, 2006). Riluzole (20 μM) alone abolished fictive sigh activity, but the fictive eupneic activity persisted (Figures 9A,B). Subsequent application of NE (20 μM, *n* = 6, Figure 9A3) did not induce sigh activity and after 5 min the rhythm completely disintegrated as previously reported (Viemari and Ramirez, 2006). We performed the same set of experiments replacing NE with isoproterenol, the β-NR agonist, after riluzole and the sigh activity never came back (Figure 9B3, *n* = 5) which support our hypothesis that *I*_Nap_ mechanisms are important for the modulation of sigh activity.

## Discussion

It is well-documented that the respiratory network simultaneously generates two distinct types of respiratory rhythms within the same anatomical area of the preBötC: “eupneic” and “sigh” rhythmic activity (Lieske et al., 2000). This raises the interesting question how the respiratory network can simultaneously generate rhythms that have two very different cycle periods. The present study revealed a fictive eupneic respiratory rhythm of ~0.18 Hz and a fictive sigh rhythm of 0.01 Hz. Several lines of evidence suggest that distinct mechanisms contribute to the generation of both rhythms. Fictive sigh rhythmic activity is exquisitely sensitive to the blockade of P/Q-type voltage-gated calcium channels and the activation of mGluR8 receptors (Lieske and Ramirez, 2006a,b). The frequency of sighs increased in the presence of NMDA blockade, while the frequency, burst duration, and regularity significantly decreased in case of the eupneic activity (Lieske and Ramirez, 2006a,b). We therefore suggested that neurons involved in the generation of sighs possess synaptic properties that are distinct from those critical for the generation of fictive eupneic activity (Lieske and Ramirez, 2006a,b). Fictive sighs and eupneic inspiratory activity are also differentially modulated by neuromodulators (Peña and Ramirez, 2002, 2004; Ramirez and Viemari, 2005; Lieske and Ramirez, 2006a,b; Viemari and Ramirez, 2006; Ruangkittisakul et al., 2008; Tryba et al., 2008). Oxotremorine, a muscarinic agonist, for example activates sighs while inhibiting fictive respiratory activity (Tryba et al., 2008). Here, we show that β-NR modulation affects specifically the sighs, but not eupneic respiratory activity, both *in vivo* and in the medullary transverse slice preparation. Although, the results obtained in the slice, are associated with caveats that are shared among many *in vitro* approaches, it is the *in vitro* approach that allowed us to further investigate the mechanisms that contribute to the generation/modulation of sigh. Our experiments explored the differential noradrenergic modulation of the two types of bursting mechanisms that have been identified in pacemaker neurons within the respiratory network: bursting that depends on the presumed activation of *I*_CAN_ (e.g., *I*_CAN_ pacemakers) and *I*_Nap_ (e.g., *I*_Nap_ pacemakers) (Thoby-Brisson and Ramirez, 2000, 2001; Peña et al., 2004; Tryba et al., 2006). We showed that β-NR activation specifically modulated bursting in *I*_*Nap*_ inspiratory pacemaker neurons, but not *I*_CAN_-dependent bursting. At the concentrations used to block the *I*_*Nap*_ current and the β-noradrenergic modulation these inspiratory neurons specifically lose their bursting properties, but continue to spontaneously generate action potentials, suggesting that it is the bursting mechanism that is required for the modulatory effect. However, it is difficult to exclude other potential mechanisms involving the *I*_*Nap*_ current. An increased *I*_*Nap*_ current will likely exert network-wide effects that may ultimately be responsible for the modulatory effect caused by the β-NR modulation. At this point we also do not know whether the bursting neurons mediate their action via a synaptic excitatory mechanism, since it has been estimated that between 10 and 50% of pacemaker preBötC neurons may be glycinergic (Morgado-Valle et al., 2010). Synaptic transmission was also not obviously affected by β-NR activation. This suggests that neither synaptic transmission nor the burst mechanism dependent on *I*_CAN_ is a critical determinant of this modulatory effect on sighs. This conclusion is further supported by the finding that FFA at the network level did neither abolish sighs, nor β-NR modulation of sighs. By contrast, β-NR modulation increased the burst frequency in *I*_*Nap*_ pacemaker neurons, neurons that rely on a *I*_*Nap*_ dependent burst mechanism. A role of *I*_*Nap*_ in modulating the sigh was also suggested by a study that investigated the effects of a muscarinic agonist oxotremorine. This agonist increased sigh frequency at the network level and specifically enhanced the frequency of a large amplitude, *I*_*Nap*_-dependent and calcium-independent burst mechanism at the level of isolated pacemaker neurons (Tryba et al., 2008).

We previously showed that riluzole applied in the presence of NE worsened the stability of fictive respiration suggesting that the presence of persistent sodium dependent mechanisms is critical for generating stable respiratory network activity (Viemari and Ramirez, 2006). However, with respect to this string of arguments it is interesting to find that β-NR modulation only affects fictive sigh production, and has no effect on the fictive eupneic rhythm. One possibility is that the modulation of the relatively fast bursting *I*_Nap_ pacemaker neurons may exert an excitatory effect on the slow sigh rhythm. This finding would be in agreement with computer modeling and experiments performed in a small crustacean network indicating that a slow network oscillator can be controlled by a much faster oscillatory neuron (Nadim et al., 1998). In the present study the effect on the fictive sigh rhythm could be derived from the activation of the bursting *I*_Nap_ pacemaker neurons. However, it must be emphasized that the mammalian respiratory network is not nearly as well-understood as the stomatogastric ganglion. Hence such a conclusion must remain largely speculative, and other mechanisms involving the persistent sodium current must also be considered.

A possible explanation for the lack of an effect on eupneic activity is that the depolarization of the *I*_Nap_-dependent pacemaker neurons was insufficient for increasing the frequency of fictive eupneic activity, and/or that additional cellular mechanisms stabilize eupneic activity against the modulation of the *I*_Nap_-dependent pacemaker neurons. Indeed, this finding would be consistent with our previously published conclusion that respiratory rhythm generation relies not only on one cellular mechanism, but that heterogeneous cellular and network properties contribute to rhythm generation and that manipulating any given mechanism alone is not sufficient to abolish rhythm generation (Peña et al., 2004). It is also consistent with the conclusion that eupnea heavily relies on synaptic mechanisms that involve *I*_CAN_ (Rubin et al., 2009).

The β-NR are known to act via a cAMP cascade, and depending on the system, cAMP can modulate a variety of ionic conductances including sodium current (Schubert et al., 1989; Kirstein et al., 1996; Weigt et al., 1998). At the pre-synaptic level in the amygdala, isoproterenol enhances excitatory transmission via β-NR (Huang et al., 1996), in the spinal cord it has been shown to increase the excitatory synaptic drive (Tartas et al., 2010). Here, we showed that isoproterenol acts on β-NR at the post-synaptic level, since isoproterenol excites *I*_Nap_ pacemaker neurons that were synaptically isolated. Moreover, β-NR did not affect excitatory synaptic transmission. Although, our finding is not incompatible with the concept that differential synaptic mechanisms are important in the generation of fictive sigh rhythm as postulated by Lieske and Ramirez (2006a,b), the present finding suggest that *I*_Nap_ pacemaker neurons could play a critical role in the modulation of sigh both *in vivo* and *in vitro* by β-NRs.

The present study has also interesting behavioral implications. It is well-established that NE plays a critical role in mediating arousal and promoting wakefulness. Its activating effects involve various subcortical areas including the locus coeruleus, the medial septal, and medial preoptic areas (Berridge et al., 2005). β-NR located within these regions seem to play a particularly important role in enhancing arousal (Berridge and Morris, 2000; Berridge, 2008). In the present study we describe that the β-NR agonist isoproterenol acts also on the preBötC, a medullary respiratory network that is critical for the generation of different forms of inspiratory activities. The sigh-specific effect of the β-NR activation is particularly remarkable, since no effects were observed on eupneic respiratory activity. This is interesting, since it is the sighs that play a critical role in the sequence of events that lead to an arousal response (McNamara et al., 1998; Wulbrand et al., 2008). The majority of spontaneous arousals during sleep occur as a stereotypic sequence that begins with a sigh which is followed by a startle and subsequent cortical arousal (McNamara et al., 2002). Similarly, in response to hypercarbia the arousal response begins with sighs (augmented breaths) that are followed by startles, thrashing limb movements, and subsequently full arousal (Thach and Lijowska, 1996; Lijowska et al., 1997). Thus, a failure to generate sighs during conditions such as hypercarbia or hypoxia may contribute to the events that eventually lead to SIDS (Peña et al., 2004). This hypothesis is supported by finding that sighs and arousals are disturbed in SIDS (Kahn et al., 1988). Previous studies also reported that the noradrenergic system is disturbed in SIDS, which includes evidence such as a diminished tyrosine hydoxylase immunoreactivity within the ventrolateral medulla of SIDS victims (Perrin et al., 1984; Obonai et al., 1998; Sawaguchi et al., 2003). Our results suggest that disturbances in noradrenergic receptors may contribute to abnormal arousal response. Our findings emphasize the need of a better understanding of the differential noradrenergic modulation of subcortical structures that have been implicated in arousal. Taken together, the β-NR of the sigh activity within the preBötC may contribute to the arousal response.

### Conflict of interest statement

The authors declare that the research was conducted in the absence of any commercial or financial relationships that could be construed as a potential conflict of interest.

## Abbreviations

preBötC: pre-Bötzinger complex
VRG: Ventral Respiratory Group.
